# Screening of Potential Plasticizer Alternatives for Their Toxic Effects on Male Germline Stem Cells

**DOI:** 10.3390/biomedicines10123217

**Published:** 2022-12-12

**Authors:** Xiangfan Zhang, Makoto Nagano

**Affiliations:** Department of Obstetrics and Gynecology, McGill University and Research Institute of the McGill University Health Centre, Montreal, QC H4A 3J1, Canada

**Keywords:** phthalate, plasticizer, toxicology, spermatogonial stem cell, compound screening, stem cell culture, spermatogonial transplantation, male fertility

## Abstract

Plasticizers give flexibility to a wide range of consumer and medical plastic products. Among them, phthalate esters are recognized as endocrine disruptors that target male reproductive functions. With this notion, past studies designed and produced alternative plasticizers that could replace phthalates with limited toxicity to the environment and to male reproductive functions. Here, we focused on one reproductive cell type that was not investigated in past studies—spermatogonial stem cells (SSCs)—and examined in vitro the effects on 22 compounds (seven plasticizers currently in use and 15 newly synthesized potential alternative plasticizers) for their effects on SSCs. Our in vitro compound screening analyses showed that a majority of the compounds examined had a limited level of toxicity to SSCs. Yet, some commercial plasticizers and their derivatives, such as DEHP (di-(2-ethylhexyl) phthalate) and MEHP (mono-(2-ethylhexyl) phthalate), were detrimental at 10^−5^ to 10^−4^ M. Among new compounds, some of maleate- and fumarate-derivatives showed toxic effects. In contrast, no detrimental effects were detected with two new compounds, BDDB (1,4 butanediol dibenzoate) and DOS (dioctyl succinate). Furthermore, SSCs that were exposed to BDDB and DOS in vitro successfully established spermatogenic colonies in testes of recipient mice after transplantation. These results demonstrate that SSC culture acts as an effective platform for toxicological tests on SSC function and provide novel information that two new compounds, BDDB and DOS, are alternative plasticizers that do not have significant negative impacts on SSC integrity.

## 1. Introduction

Phthalates are widely used plasticizing compounds that give flexibility and durability to plastic products, including household items and medical devices [1,2,3,4,5]. DEHP (di-(2-ethylhexyl) phthalate) is one of such compounds that is commonly used to provide plasticity to polyvinyl chloride (PVC) [6]. As a common property of plasticizers, phthalates are not covalently bound to plastic matrices, such as PVC, and thus can leach out to the environment. Consequently, phthalates are known to be environmental contaminants [4], and their anti-androgenic properties have become a problem for the reproductive health of males [2,7,8]. In fact, many animal studies have reported that the exposure to phthalates can disrupt testosterone secretion and development of testicular germ and somatic cells [9,10,11,12,13].

A series of recent studies sought newly developed, alternative plasticizers, which have a comparable plasticizing ability to phthalates, but less negative impacts to environmental and biological systems [1,3,14,15,16,17,18,19,20,21]. These studies evaluated 15 newly synthesized compounds and compared their effects on reproductive cells with those of seven commercial plasticizers (Table 1). These studies generated important information about toxicological effects on major testicular cell types using immortalized cell lines (MA10 cells for Leydig cells, TM4 cells for Sertoli cells, and C18-4 for spermatogonia) and identified alternative plasticizers, which were called ‘green plasticizers,’ to replace phthalates. However, the effects on male germline stem cells, or spermatogonial stem cells (SSCs), were not examined, even though these cells are the fundamental resource to maintain the health of our future population and can be a vehicle to transmit toxicological consequences to subsequent generations [3,4,22]. These studies used the C18-4 cell line as a model of undifferentiated spermatogonia [14,21,23], but these cells have not been demonstrated for their ability to induce spermatogenesis and produce spermatozoa. In order to derive toxicological data on SSCs, primary cells that can regenerate spermatogenesis are the most optimal as a test cell population.

By definition, stem cells are the cells that regenerate an entire cell lineage and maintain its steady-state homeostasis for a lifetime [24]. For SSCs, the gold standard for detecting the ability to regenerate spermatogenesis has been spermatogonial transplantation in the mouse model [25,26,27]. In this technique, a single-cell preparation of donor cells is injected into the seminiferous tubules of recipients, and SSCs included in the donor cell preparation engraft and re-establish complete spermatogenesis in the form of the segments of seminiferous tubules, called “colonies.” The number of colonies observed in recipient testes represent the number of SSCs that successfully regenerated donor-derived spermatogenesis upon transplantation [28]. Importantly, mouse SSCs can be maintained in vitro for a long time while their regenerative capacity is sustained. Cultured SSCs thus can establish colonies of donor-derived spermatogenesis after transplantation at any time during the long-term culture period [29,30,31]. This SSC culture technique allows us to examine faithfully the effects of various compounds, toxic materials, or growth factors on SSCs. 

**Figure 1:**
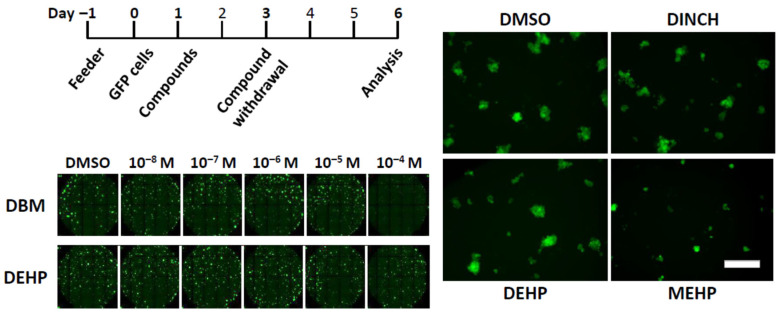
Automated image capture of clusters after short-term exposure to plasticizers. (**Top Left**) Culture schedule of the short-term exposure scheme. Feeder layers were prepared a day before placing GFP cluster cells in culture, while compounds were added a day after cell seeding. Compounds were withdrawn on day 3 and media and growth factors are replenished. Images were acquired on day 6. (**Bottom Left**) Representative composite image of cluster cultures exposed to different ranges of compound concentrations. Each dot with green fluorescent signal represents one cluster. DBM and DEHP are shown here as examples, which were added at the concentration of 10^−8^ M to 10^−4^ M. (**Right**) A panel of representative image of GFP clusters exposed to DMSO, DINCH, DEHP, or MEHP at 10^−4^ M. Scale bar = 20 µm.

When cultured, SSCs proliferate while producing committed daughter cells, resulting in the formation of aggregates or islands of undifferentiated spermatogonia, which we call “clusters,” within 6 days in vitro [31,32] (Figure 1). It has been demonstrated that the number of clusters linearly correlates with the number of SSCs that survived in vitro while the area or the size of clusters reflects the ability of SSCs to produce progenitor spermatogonia (immediate descendants of SSCs) as well as the activity of progenitors to proliferate [31,32,33]. Compared to spermatogonial transplantation, which requires two months to acquire data [27], the simplicity of the in vitro SSC culture provides us with an important tool to assess the effects of a number of chemicals and compounds on SSC activity in a short period of time based on the cluster forming activity: i.e., cluster numbers and size. In fact, we previously used this “cluster-forming assay” and reported, combining it with an unbiased computer-assisted image analysis, the detrimental effects of chemotherapeutic compounds on SSCs [32,33]. In this study, we took advantage of our expertise in the cluster-forming assay and spermatogonial transplantation and assessed the effects of newly synthesized plasticizing compounds on SSCs in the mouse model to identify an alternative plasticizer that is safe for SSCs. 

## 2. Materials and Methods

### 2.1. Plasticizing Compounds

A total of 22 compounds (commercial plasticizers and potential alternatives) were examined in this study. Five compounds (DEHP, MEHP, DEHA (diethylhexyl adipate), DEHM (diethylhexyl maleate), and DBM (dibutyl maleate)) were purchased from Sigma Aldrich (St-Louis, MO, USA), while DINCH (1,2-cyclohexane dicarboxylic acid di-isononyl ester) was from BASF Canada Inc. (Missisauga, ON, Canada) (Table 1). All other compounds were synthesized, with a purity of ≥99%, as previously described [34,35,36]. These synthesized compounds belong to four chemical families: dibenzoates, succinates, maleates, and fumarates [1,14]. The stock solution of each compound was prepared in dimethylsulfoxide (DMSO, Sigma Aldrich) and then diluted with DMSO to the desired concentrations prior to use. The final DMSO concentration in the medium of all plasticizer assays was 0.1% (*v/v*). Control groups received 0.1% DMSO only.

### 2.2. Animals

GFP mice (C57BL/6-Tg(CAG-EGFP)1Osb/J; Jackson Laboratory) express GFP ubiquitously [37]. Male pups of this mouse strain on postnatal days 6–8 were used to induce primary culture of undifferentiated spermatogonia, including SSCs (see below), as in [31]. Recipient mice for spermatogonial transplantation were 129/SvEv x C57BL/6 (B6) F1 males treated with 50 mg/kg of busulfan 4–6 weeks before transplantation [27]. All procedures for animal handling and care were approved by the Animal Care and Use Committee at the Research Institute of the McGill University Health Centre. 

### 2.3. Cell Culture

SSCs culture was performed as described previously [31,32,33]. In short, Thy1-positive testis cells were isolated from B6GFP males at 6–8 days postpartum (dpp) and enriched for SSCs using an immunomagnetic cell sorting apparatus (Dynal). Sorted cells were placed in 24-well plates on a feeder layer of mitotically inactivated STO (SIM mouse embryo-derived thioguanine- and ouabain-resistant) cells (a feeder layer) using mitomycin C. Thy1-positive cells were cultured at 37 °C with 5% CO_2_ in a serum-free medium based on MEMα medium supplemented with 40 ng/mL GDNF (R&D systems), 300 ng/mL GDNF family receptor α1 (GFRα1; R&D systems), and 1 ng/mL fibroblast growth factor 2 (FGF2; Life technologies), as in [31]. Under these conditions, Thy1-positive cells proliferate and form aggregates or ‘islands’ of undifferentiated spermatogonia, including SSCs [31]; these aggregates are termed “clusters” in this study. Clusters emerged by day 6 after seeding. Media were replenished on day 3 in vitro, and clusters were subcultured onto freshly prepared STO feeder cells with a 6–7-day interval [31]. Once established (after five passage generations), no donor-derived somatic cells remain in culture, and clusters can be maintained for a long time with reduced concentrations of growth factors: 20 ng/mL GDNF, 75 ng/mL GFRα1, and 1 ng/mL FGF2 [31]. The compound screening experiments were conducted using established clusters with the reduced concentrations of growth factors. 

### 2.4. Compound-Screening Experiments

STO feeder cells were prepared a day before experiments throughout this study. For the short-term screening experiments, 2500 single GFP cluster cells were placed in the black-wall flat/clean-bottom half-area 96-microwell plates (CLS 3882, Corning Inc., New York, NY, USA). The cells were incubated in 80 µL of the serum-free medium with growth factors at reduced concentrations as described above; the day of cluster cell seeding was defined to be day 0 in vitro. Next day, 20 µL of the compounds at diverse concentrations or vehicle (DMSO, 0.1% *v/v*) were added to cluster cultures; thus, the final medium volume was 0.1 mL/well during the experimental period. Two days after, culture media were changed to fresh media and growth factors with no compounds or DMSO. Cultures continued for additional 3 days; thus, cluster cells were analyzed on day 6. 

For long-term experiments, compounds were added to the cluster culture on day 1 and incubated with clusters for 6 days. Media and growth factors were replenished on day 4 during the culture period, and clusters analysed on day 7.

To test the effects of compounds on the feeder layer, STO feeders were cultured in serum-free media with growth factors and select compounds for 6 days, with the media, growth factors, and compounds replenished on day 3. On day 6, STO feeder layer was washed with PBS, and GFP cluster cells (2500 cells) were placed on these treated STO feeders, followed by analyses 3 days later.

For long-term experiments, compounds were added into cluster culture on day 1 and incubated with clusters for 6 days. Media and growth factors were replenished on day 4 during the culture period, and clusters analysed on day 7. 

To test the effects of compounds on the feeder layer, STO feeders were cultured in serum-free media with growth factors and select compounds for 6 days, with the media, growth factors, and compounds replenished on day 3. On day 6, STO feeder layer was washed with PBS, and GFP cluster cells (2500 cells) were placed on these treated STO feeders, followed by analyses 3 days later. 

### 2.5. Image Acquisition and Cluster Analysis

Automated image acquisition and analyses were conducted as described previously using the ImageXpress^micro^ high content imaging system (Molecular Devices, Sunnyvale, CA) [33]. The system is equipped with an incubator to maintain a constant temperature, carbon dioxide concentration, and humidity during analyses. On the day of analysis at the end of a designated culture period, culture plates were transferred to our institutional imaging facility and placed in the incubator of the image capture apparatus. For each well of the plates, 30 pictures were acquired; the incubator was set to 37 °C and maintained at 5% CO_2_ during the procedure. Thereafter, MetaXpress software (Molecular Devices) was used to determine the number and surface area of clusters. Guided by GFP signals, clusters were defined as objects with the minimum width of 30 µm and the maximum width of 180 µm, which correspond to the aggregates of at least six cells [33]. Cluster surface area was defined as the total area of all pixels inside projected perimeter of cluster that have fluorescence intensity above the set threshold value (local background 150 gray levels). Cluster numbers represent those of SSCs that survived while cluster area reflects the activity of SSCs to produce committed progenitors and the ability of progenitors to proliferate during the exposure to compounds [33]. 

### 2.6. Spermatogonial Transplantation

Cluster cells were transplanted as previously described [27,31]. Briefly, at the end of the plasticizer treatment, cultured cells were harvested as single cells by trypsin digestion and re-suspended in an injection medium (DMEM with 10% FBS, 0.1 mg/mL DNase, and 0.04% trypan blue). Approximately 7 µL of 1.0 × 10^6^ cells/mL were injected into each recipient testis via the efferent tubules, resulting in 75–80% filling of the tubules [27]. Two months after transplantation, the recipient mice were euthanized, their testes collected, and GFP-positive colonies of donor-derived spermatogenesis quantified under a fluorescence stereomicroscope. The colony number reflects the number of functional SSCs that engrafted after transplantation in a sample of cultured cells [28].

### 2.7. Statistics

All data are presented as the mean ± SEM. Statistical analysis was performed using ANOVA followed by Fisher’s Least Significant Difference multiple comparisons. Differences were considered significant when *p* < 0.05. 

## 3. Results

The compounds tested are listed on Table 1. In addition to seven commercial plasticizers, four groups of newly designed chemicals were tested in this study as alternative plasticizers: derivatives of dibenzoates, maleates, succinates, and fumarates. These compounds are structurally similar to DEHP, and their plasticizing properties and environmental toxicity have been examined [1,16,17]. In addition, past studies evaluated the toxicological effects of the same compounds on male reproductive cells [3,4,14,15,16,17,18,19,20,21]. In this study, we screened these compounds in vitro for their effects on mouse SSCs and undifferentiated spermatogonia using the cluster-forming assay [31,32]. We established spermatogonial clusters from GFP transgenic mice, which express GFP ubiquitously [37], such that the clusters can be detected and measured in real time based on the fluorescent signal, and the number and size of clusters can be determined in an unbiased manner using a computer-assisted image analysis technique [33] (Figure 1). 

### 3.1. Acute Exposure to Maleate and Fumarate Derivatives Negatively Impacts Undifferentiated Spermatogonia In Vitro

We first examined acute effects of compounds on mouse SSCs after a 2-day exposure (Figure 1). We used this experimental paradigm in order to expose SSCs immediately after they were released from spermatogonial aggregations, or “clusters” [31,33] (Figure 1 right). The rationale of this paradigm was that in a few days after passaging, cluster cells generate three-dimensional structure (cell island) because of the accumulating progenitor cells produced by SSCs, thereby potentially encapsulating SSCs within a cluster and interfering with the access of a compound to SSCs. 

Cluster cells were placed on feeder cells in culture. On the next day, compounds were added at doses ranging from 10^−8^ to 10^−4^ M, dissolved in DMSO (Figure 1). Two days later, the medium was replaced with a fresh medium without compounds, and clusters were allowed to grow for an additional 3 days under our standard cluster culture condition. On day 6, the number and size of clusters were measured using a computer-assisted image analysis apparatus (Figure 1). As experimental readouts, we focused on cluster numbers and cluster size. As described above, the cluster number indicates the quantity of SSCs (i.e., SSC survival), while the cluster size reflects SSC commitment to differentiation (i.e., progenitor production and proliferation) under the exposure to a compound for two days. 

**Figure 2:**
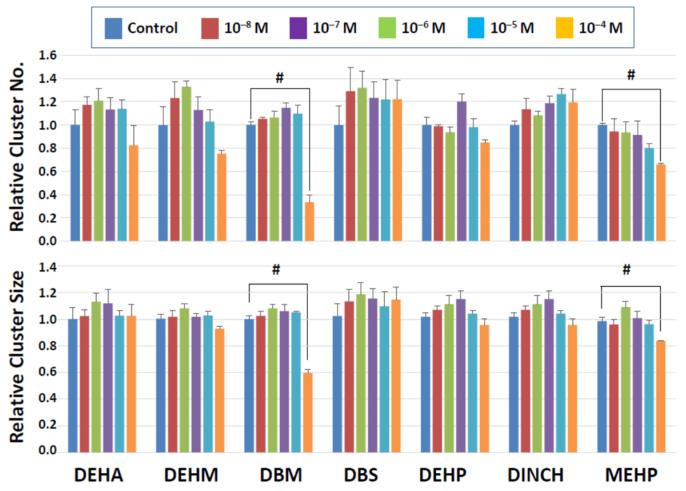
Cluster numbers and sizes after 2-day exposure to commercial plasticizers. The data are normalized to the results of control (DMSO) treatment. Hash signs (#) indicate significant differences (*p* < 0.05).

Figure 2 shows the results of 2-day exposure of clusters to seven commercial plasticizers. The results indicate that a majority of commercial plasticizers tested neither showed significant effects on cluster numbers nor size at any doses used. Two compounds, MEHP, an active metabolite of DEHP, and DBM, however, reduced the number and size of clusters. Although a trend was seen with MEHP in that the cluster number declined in a dose-dependent manner, a significant decrease was detected only at the highest dose (10^−4^ M). A negative impact of MEHP was also observed on cluster size at 10^−4^ M. Likewise, DBM at 10^−4^ M markedly reduced the cluster number and size. These results suggest that the negative impact of commercial plasticizers on cluster-forming activity after two days of exposure was relatively limited, but MEHP and DBM showed detrimental effects at the highest concentration tested even after a mere 2-day exposure. 

**Figure 3:**
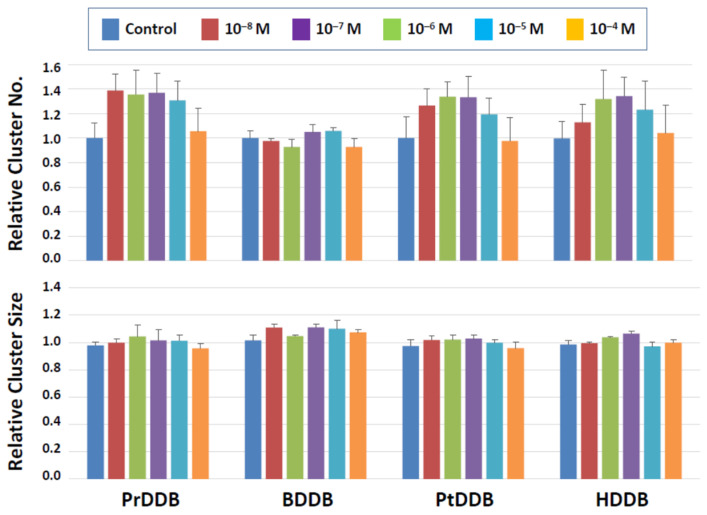
Cluster numbers and sizes after a 2-day exposure to dibenzoate derivatives (Figure 3). The data are normalized to the results of control (DMSO) treatment. No detrimental effects are detected on the cluster-forming activity in the presence of these two types of compounds.

**Figure 4:**
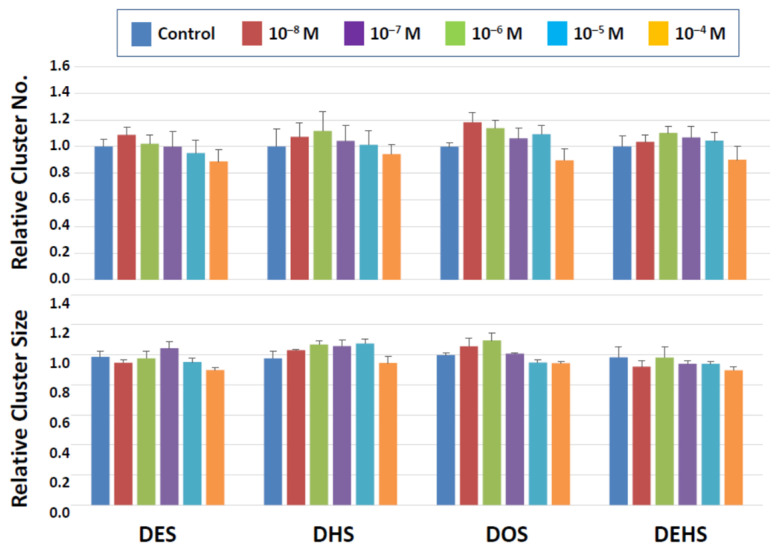
Cluster numbers and sizes after a 2-day exposure to succinate derivatives. The data are normalized to the results of control (DMSO) treatment. No detrimental effects are detected on the cluster-forming activity in the presence of these two types of compounds.

Among the four categories of newly synthesized potential plasticizing compounds (derivatives of dibenzoates, maleates, succinates, and fumarates), none of those that belong to the dibenzoate and succinate groups showed detrimental effects (Figure 3 and Figure 4). Some dibenzoate derivatives (PrDDB, PtDDB, and HDDB) appeared to increase the cluster number, but no significance was detected (Figure 3). Cluster size in the dibenzoate group (Figure 3) as well as both cluster number and size in the succinate derivatives (Figure 4) remained similar to the level observed after exposure to the vehicle (DMSO).

**Figure 5:**
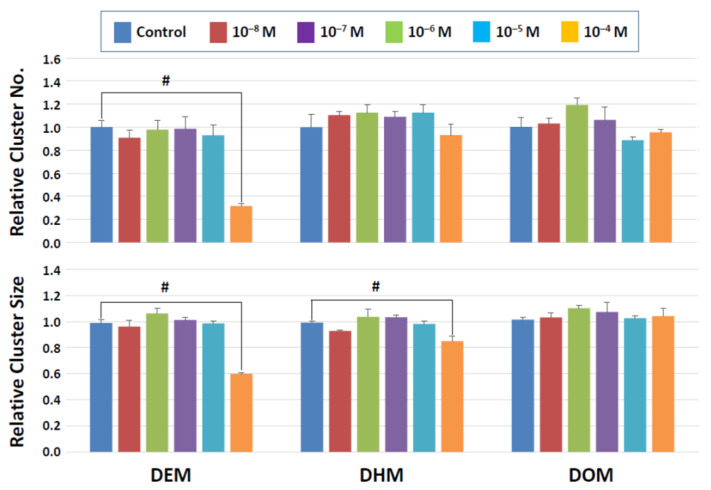
Cluster numbers and sizes after a 2-day exposure to maleate derivatives. The data are normalized to the results of control (DMSO) treatment. Hash signs (#) denote significant differences (*p* < 0.05). A significant reduction in cluster numbers is seen with DEM between control and 10^−4^ M whereas cluster sizes decline in the presence of 10^−4^ M DEM and DEH.

**Figure 6:**
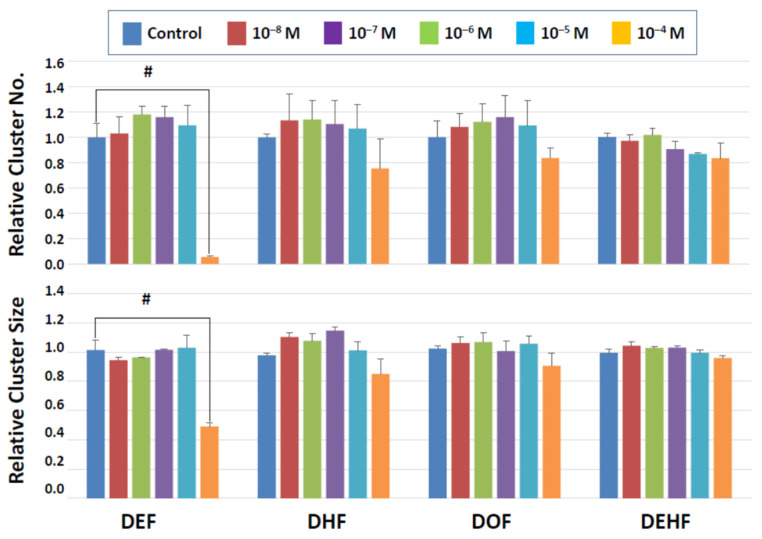
Cluster numbers and sizes after a 2-day exposure to fumarate derivatives. The data are normalized to the results of control (DMSO) treatment. Hash signs (#) denote significant differences (*p* < 0.05). A significant reduction in cluster number and cluster size is detectable at 10^−4^ M DEF.

On the other hand, significant detrimental effects were seen with the maleate and fumarate derivatives at the highest concentration tested (10^−4^ M). In the maleate group (Figure 5), DEM significantly reduced both the cluster number and size, suggesting that SSC numbers declined, and the production of SSC descendants was negatively impacted. DHM showed a statistically significant effect only on the cluster size at 10^−4^ M, implying that this compound did not affect SSC survival but may have negatively affected the ability of SSCs to produce daughter cells. In the fumarate group (Figure 6), DEF reduced both the cluster number and size at 10^−4^ M. Overall, this short-term exposure scheme revealed that dibenzoate and succinate derivatives are preferable plasticizers compared to those derived from maleates and fumarates when the toxicity to SSCs is considered. 

### 3.2. Long-Term Exposure Confirms the Minimal Adverse Effects of Alternative Plasticizers

**Figure 7:**
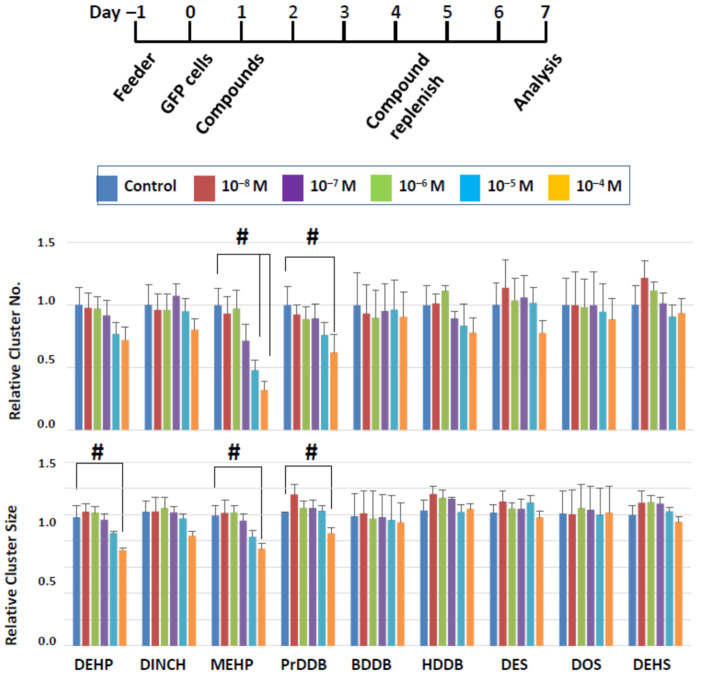
Cluster numbers and sizes after a 6-day exposure to select plasticizing compounds. The data are normalized to the results of control (DMSO) treatment. Hash signs (#) denote significant differences (*p* < 0.05). A significant reduction in cluster number and size is seen when clusters were treated with MEHP or PrDDB, while DEHP showed a negative impact on cluster size alone.

The results of short-term exposure generally indicated that negative effects of the compounds on cluster forming activity was limited. It is possible that cluster cells tolerated the compound actions because of the short exposure time of two days and also because cluster cells were cultured in our standard condition from day 3 with no compound actions imposed. We therefore exposed clusters to the compounds continuously for six days (Figure 7); we routinely maintain cluster culture with a 6-day interval of passaging during long-term SSC culture [29,30,31]. We focused on dibenzoate and succinate derivatives in this series of experiments for three reasons. First, we did not observe detrimental effects with dibenzoate and succinate derivatives while at least one compound of maleates and fumarates were detrimental to cluster formation. Second, past studies indicated that the plasticizer property of fumarate derivatives were less robust compared to dibenzoates or succinates [1,16,17]. Finally, maleates were reported to be detrimental to a mouse Leydig cell line [18]. Thus, we selected three compounds each from dibenzoate and succinate derivatives, as well as three commercial plasticizers (DEHP, MEHP, and DINCH) as a comparison group. Clusters were incubated with the compounds for 6 days, during which the medium was changed once with freshly added growth factors and compounds from 10^−8^ to 10^−4^ M (Figure 7); cluster numbers and size were measured after the completion of the 6-day exposure (i.e., on day 7 in vitro).

In the commercial plasticizer group, all three compounds tended to reduce the number and size of clusters in a dose-dependent manner, yet significant effects were detected only with MEHP and DEHP and not with DINCH (Figure 7). MEHP significantly reduced the cluster number at 10^−5^ and 10^−4^ M and the cluster size at 10^−4^ M. In this experimental approach, the cluster number reflects the number of surviving SSCs, as in the short-term approach, but the cluster size can reflect the combined effects of a compound on the ability of SSCs to produce daughter cells as well as the ability of daughter cells to proliferate in the presence of the chemical compounds. Thus, both SSCs and their descendants may be affected by MEHP. Although DEHP did not show negative effects in the acute experiment, it significantly reduced the cluster size after a 6-day exposure at 10^−4^ M, with no effects on cluster numbers, suggesting that DEHP at a high dose may have more detrimental effects on progenitor proliferation, rather than on SSC survival. 

Most of the novel compounds did not affect the cluster growth, but PrDDB, which did not show an effect after a 2-day exposure, negatively affected both the number and size of clusters at 10^−4^ M. These results indicate that a longer exposure to plasticizers can increase the negative impact on SSCs and suggest that PrDDB is not a desirable candidate as a green plasticizer.

**Figure 8:**
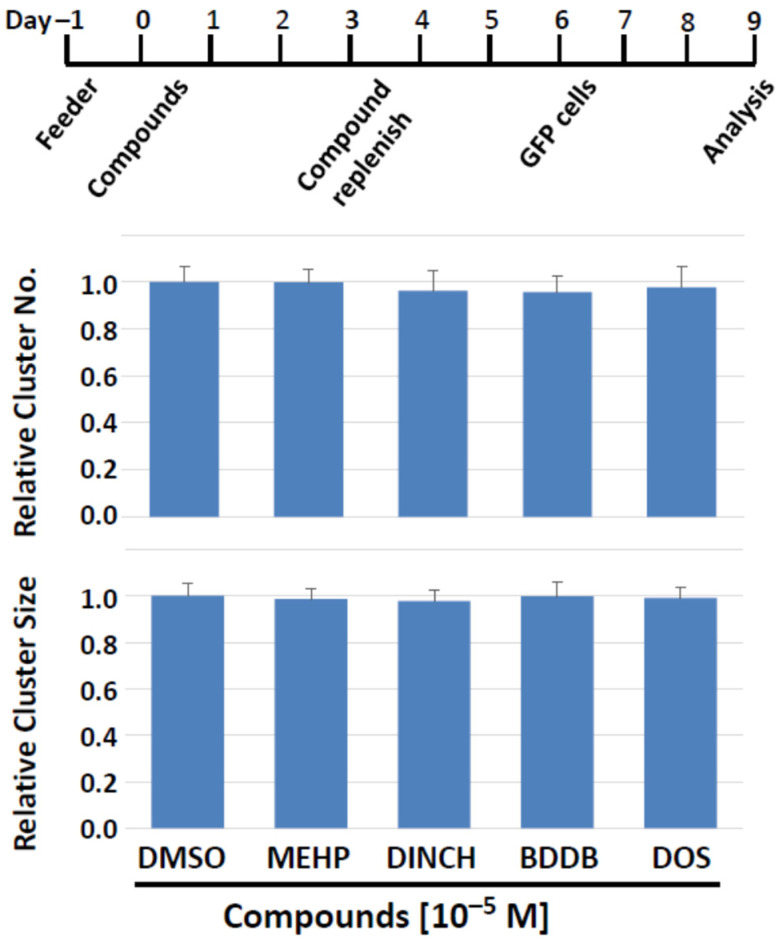
Feeder cells pretreatment with plasticizing compounds for 6 days can still support normal cluster forming activity. (**Top**) The treatment schedule. On days 3 in vitro, media and compounds were replenished. Cluster cells were seeded on treated feeder cells on day 6, and their number and size were determined on day 9. (**Bottom**) Number and size of clusters. Feeder cells that experienced potentially negative effects of test compounds do not lose their ability to support cluster formation.

Our cluster-forming assay employs feeder cells to support the development of clusters. It is thus possible that the compounds may affect the activity of feeder cells, thereby indirectly modulating SSC activity. Since feeder cells are an indispensable component of successful SSC culture, it is difficult to distinguish the effects of compounds on feeder cells from those on cluster cells. Therefore, we first exposed the feeder cells alone to compounds for 6 days, as shown in Figure 7, and then placed a single-cell suspension of cluster cells on the feeder cells pre-treated with compounds. Thereafter, cluster culture was performed under our standard protocol for the subsequent 3 days to induce cluster formation (Figure 8); cluster formation can be first recognized 3 days after seeding the cells in vitro. Finally, we measured the number and size of spermatogonia clusters. 

As target compounds, we chose MEHP, DINCH, BDDB, and DOS, for the following rationales. MEHP showed significant negative effects on cluster growth in both 2-day and 6-day exposure schemes. DINCH is a commercial plasticizer that did not exhibit detrimental effects in screenings experiments. BDDB and DOS were selected because they did not exhibit negative impact on cluster formation, while they have been shown to have plasticizing ability comparable to the plasticizers currently in use [1,16,17]. In addition, BDDB and DOS have lower or equivalent levels of leaching and exhibit the least toxicity to bacteria and a mammalian cell line [1,16,17]. 

The results showed that none of the compounds affected the two parameters of cluster growth (Figure 8). Importantly, this was also the case when MEHP, the most detrimental compound among those tested, was applied. Therefore, the plasticizer compounds tested apparently do not have prominent detrimental effects on the cluster-supporting function of feeder cells, and the effects on cluster cells observed in this study are suggested to be direct on cluster cells, including SSCs. 

### 3.3. BDDB and DOS Allows for Normal SSC Engraftment after Transplantation

**Figure 9:**
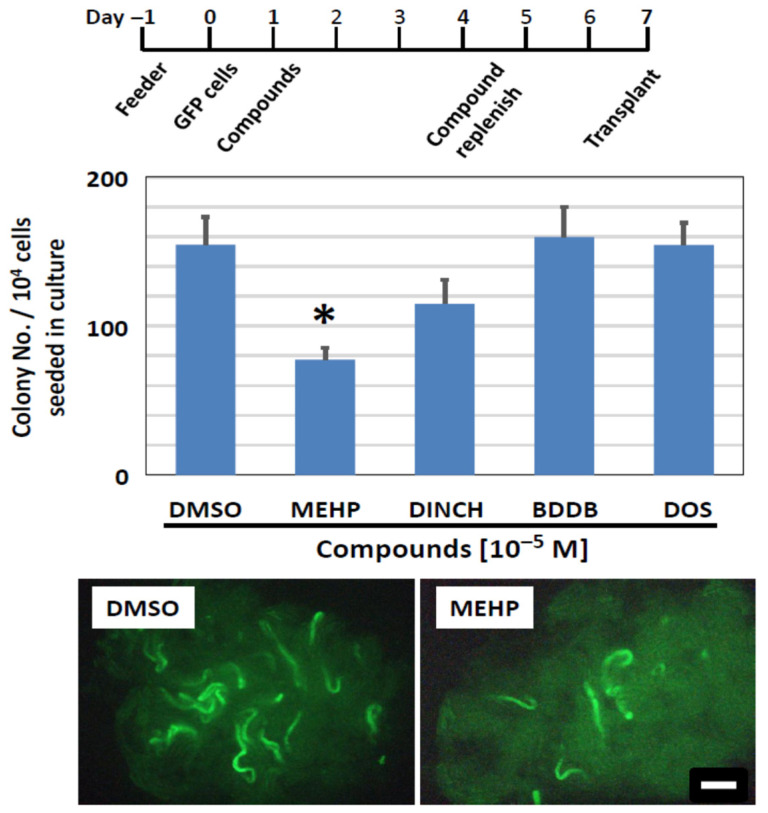
Colonization ability of SSCs exposed to plasticizing compounds, measured using spermatogonial transplantation. (**Top**) Schedule of compound exposure. It followed the 6-day exposure protocol as shown in Figure 7. (**Middle**) Results of the transplantation assay. Newly synthesized compounds, BDDB and DOS, did not affect the colonization ability of SSCs, as the number of spermatogenic colonies established after transplantation is comparable to that of control (DMSO). Although DINCH shows a trend of a reduced colony number, the difference was not significant. MEHP significantly reduces the colony number (Asterisk). (**Bottom**) Photomicrographs of recipient testes transplanted with GFP cluster cells, including SSCs, that were exposed to DMSO or MEHP. Green stretches of seminiferous tubules indicate the colony of spermatogenesis derived from cultured SSCs. Scale bar = 1 mm.

Following the series of in vitro screening experiments as above, we evaluated the in vivo engraftment capacity of SSCs that were exposed to plasticizing compounds in vitro, using spermatogonial transplantation. Clusters were first treated with MEHP, DINCH, BDDB, or DOS in vitro at 10^−5^ M for 6 days (Figure 9, top). They were then harvested and transplanted into recipient mouse testes. Two months later, regeneration of spermatogenesis derived from treated SSCs was confirmed by detecting GFP-positive colonies along the recipient seminiferous tubules (Figure 9, bottom), as reported in [29,31,38]. The number of colonies was determined visually under a fluorescent stereomicroscope. Colony numbers indicate the number of SSCs that successfully colonized and regenerated spermatogenesis after transplantation [27,32]. We found that MEHP significantly reduced the number of colonies to 50% of the control level (Figure 9), confirming the results of the cluster-forming assay in vitro (Figure 7). Following exposure to DINCH, there appeared to be a level of reduction in colony numbers, but no significance was detected. Importantly, the number of spermatogenic colonies after exposure to BDDB and DOS was equivalent to the control level, indicating that these compounds did not negatively affect the survival and engraftment capacity of SSCs after exposure in vitro. These results confirm the results obtained in vitro and suggest that MEHP is detrimental to SSCs, while the novel plasticizers, BDDB and DOS, are safe compounds to sustain the regenerative activity of SSCs under the condition examined in this study. 

## 4. Discussion

In this study, we screened potential plasticizing compounds with an aim to determine their direct toxicity on SSCs. SSC function is unequivocally determined using spermatogonial transplantation, but this technique is so labor-intensive and time-consuming that it is not amenable to screen many compounds at once, as aimed at in this study. We therefore took an in vitro approach based on the cluster-forming assay, together with the unbiased data acquisition using computer-assisted image analyses. This approach allowed us to efficiently test biological effects of alternative plasticizers for their effects on SSCs. Importantly, the cluster-forming assay helped us to reduce the number of compounds to be tested for their in vivo colonization ability using spermatogonial transplantation. The data obtained in the in vitro assays were confirmed by spermatogonial transplantation (Figure 9), attesting to the fidelity of the in vitro approach based on the cluster-forming assay to assess the SSC toxicity. The results obtained in this study indicate that BDDB and DOS, two compounds which have been reported to be readily degraded by soil bacteria with appreciable plasticizing ability [1,16,17], can be effective plasticizers that showed no detectable toxicity on SSCs. Our study also provides supportive evidence for a recent in vivo toxicological study that reported BDDB and DOS to be viable ‘green’ plasticizers [15].

A previous study investigated the germ cell toxicity of the same array of compounds as tested in this study using a spermatogonial cell line, C18-4 [14]. These cells express multiple genes that are associated with undifferentiated spermatogonia and thus have been a useful tool to study spermatogonia [23,39]. The study determined the toxicity based on two parameters: the cell viability, as measured by the MTT assay, and the cell replication by BrdU incorporation [14]. The results showed that all maleate derivatives significantly reduced both viability and replication activity of C18-4 cells while fumarate derivatives were also found to be detrimental. As such, the results of our study generally tend to agree with those of the results obtained using C18-4. However, some noted differences can also be identified. For example, a benzoate derivative (HDDB) and a succinate derivative (DEHS) were detrimental to C18-4 cells, but we did not observe such effects on cluster forming activity (Figure 3, Figure 4 and Figure 7). Importantly, MEHP, which showed the most prominent negative effects on cluster formation and colonization capacity of SSCs, did not show adverse effects on C18-4 cells. These observations suggest that the use of naïve SSCs is ideal to understand the toxicological effects on the specific target cell type.

While a great majority of succinate or dibenzoate derivatives did not show negative effects on numbers and sizes of clusters, such effects were detectable with maleate- and fumarate-derivatives (Figure 2, Figure 3, Figure 4, Figure 5 and Figure 6). Boisvert et al. [14] reported that maleate- and fumarate-derivatives were detrimental for survival, proliferation, and steroid production of the MA-10 Leydig cell line. Maleate derivatives are also reported to reduce the viability of Sertoli cell lines (MSC-1 and 15p-1) [40]. Together with our study, these results indicate that maleate- and fumarate-derivatives have negative impact in testicular germ and somatic cells and thus are not preferable alternative plasticizers. 

Among the plasticizers currently on market, we detected the SSC toxicity in MEHP, which is an active metabolite of DEHP but is not a plasticizer in itself. We also note that while DEHP did not show the toxicity after the short-term exposure, its longer exposure caused a decline in cluster size without affecting cluster numbers (Figure 2 and Figure 7). This is suggestive that DEHP may act more on descendants of SSCs and adversely affect their proliferation, thereby reducing the cluster size. It appears therefore that DEHP may exert SSC toxicity by itself but is more likely to show a prominent negative impact as a metabolite. On the other hand, another commercial plasticizer, DINCH, did not show toxicity on SSCs in our study. 

In this context, MEHP reduced both the number and size of clusters in both short- and longer-term in vitro assay. In addition to reducing the survival of SSCs, therefore, this compound also exerts its negative impact on the cells’ ability to produce committed progenitors as well as on the proliferation ability of committed germ cells. These observations derived in vitro complement the results of transplantation assay, where MEHP showed a significant decline in the regeneration of activity of SSCs after transplantation. 

In general, negative effects were observed only at high concentrations. For instance, MEHP caused reduced cluster numbers and size after the 6-day exposure only at 10^−4^ and 10^−5^ M. According to a previous study [41], serum levels of DEHP and MEHP in humans are 5.9 ng/mL and 0.77 ng/mL on average, respectively; the maximum level reported was 129 ng/mL for DEHP and 4.5 ng/mL for MEHP. These levels correspond to the average of 1.5 × 10^−8^ M and 0.2 × 10^−8^ M, respectively. Therefore, the doses of compounds that led to the SSC toxicity in this study were much higher than those recorded in humans. This may imply that phthalates are in general not directly toxic to SSCs or that SSCs may be robust cells and are capable of tolerating toxic insults by phthalates, at least after an in vitro exposure as tested here. However, in view of reports that exposure to phthalate in vivo can result in transgenerational effects on the phenotypes of offspring [3,22], further investigations, such as epigenetic and transcriptome analyses as well as the combined use of multiple chemicals in the cluster-forming assay, are needed to establish the SSC toxicity of plasticizing compounds. This is particularly the case considering that the exposure of SSCs to phthalates or plasticizers are a continuous event from the time of conception in the mother’s womb to after birth throughout the life. In this perspective, it is certainly ideal to carry out compound screening using human SSC in vitro. The establishment of human SSC culture is essential for toxicological analyses. 

In this study, we demonstrated that SSC culture provides an effective approach for compound screening to examine SSC toxicity. The use of primary cells that have regenerative capacity is the unique strength of this approach that cannot be expected from the use of immortalized cell lines. This in vitro analysis should promptly identify target compounds for the next step of toxicological studies, in vivo testing using live animals, which is more time-consuming and labor-intensive but more physiologically relevant. 

## 5. Conclusions

The long-term SSC culture system provides an effective platform to screen compounds and toxins for clinical and toxicological investigations. The current study examined the negative impact of potential “green plasticizers” on SSCs and identified two compounds, BDDB and DOS, that represent next-generation plasticizers which have minimal detrimental effects on the regenerative capacity of SSCs.

## Figures and Tables

**Table 1:** A list of compounds tested in this study.

Plasticizer Tested	Abbreviation
**<Commercial Plasticizers>**	
Di-(2-ethylhexyl) phthalate	DEHP
Mono-(2-ethylhexyl) phthalate	MEHP
1,2-Cyclohexane dicarboxylic acid, di-isononyl ester	DINCH
Diethylhexyl adipate	DEHA
Diethylhexyl maleate	DEHM
Dibutyl maleate	DBM
Dibuthyl succinate	DBS
**<Dibenzoate>**	
1,3 Propanediol dibenzoate	PrDDB
1,4 Butanediol dibenzoate	BDDB
1,5 Pentanediol dibenzoate	PtDDB
1,6 Hexanediol dibenzoate	HDDB
**<Succinate>**	
Diethyl succinate	DES
Dihexyl succinate	DHS
Dioctyl succinate	DOS
Di(2-ethylhexyl) succinate	DEHS
**<Maleate>**	
Diethyl maleate	DEM
Dihexyl maleate	DHM
Dioctyl maleate	DOM
**<Fumarate>**	
Diethyl fumarate	DEF
Dihexyl fumarate	DHF
Dioctyl fumarate	DOF
Di(2-ethylhexyl) fumarate	DEHF

## Data Availability

The data presented in this study are available on request from the corresponding author.
